# Preparation and optimization of matrix metalloproteinase-1-loaded poly(lactide-*co*-glycolide-*co*-caprolactone) nanoparticles with rotatable central composite design and response surface methodology

**DOI:** 10.1186/1556-276X-7-359

**Published:** 2012-07-02

**Authors:** Ping Sun, Hua Song, Daxiang Cui, Jun Qi, Mousheng Xu, Hongquan Geng

**Affiliations:** 1 Department of Pediatric Urology, Xinhua Hospital, Shanghai Jiao Tong University School of Medicine, 1665 KongJiang Road, Shanghai, 200092, People's Republic of China; 2 Department of Bio-Nano Science and Engineering, Key Laboratory for Thin Film and Microfabrication of Ministry of Education, Institute of Micro-Nano Science and Technology, Shanghai Jiao Tong University, 800 Dongchuan Road, Shanghai, 200240, People's Republic of China; 3 Department of Urology, Xinhua Hospital, Shanghai Jiao Tong University School of Medicine, 1665 KongJiang Road, Shanghai, 200092, People's Republic of China

**Keywords:** Matrix metalloproteinase-1, PLGA-PCL, Nanoparticles, Rotatable central composite design, Response surface methodology

## Abstract

Matrix metalloproteases are key regulatory molecules in the breakdown of extracellular matrix and in inflammatory processes. Matrix metalloproteinase-1 (MMP-1) can significantly enhance muscle regeneration by promoting the formation of myofibers and degenerating the fibrous tissue. Herein, we prepared novel MMP-1-loaded poly(lactide-co-glycolide-co-caprolactone) (PLGA-PCL) nanoparticles (NPs) capable of sustained release of MMP-1. We established quadratic equations as mathematical models and employed rotatable central composite design and response surface methodology to optimize the preparation procedure of the NPs. Then, characterization of the optimized NPs with respect to particle size distribution, particle morphology, drug encapsulation efficiency, MMP-1 activity assay and *in vitro* release of MMP-1 from NPs was carried out. The results of mathematical modeling show that the optimal conditions for the preparation of MMP-1-loaded NPs were as follows: 7 min for the duration time of homogenization, 4.5 krpm for the agitation speed of homogenization and 0.4 for the volume ratio of organic solvent phase to external aqueous phase. The entrapment efficiency and the average particle size of the NPs were 38.75 ± 4.74% and 322.7 ± 18.1 nm, respectively. Further scanning electron microscopy image shows that the NPs have a smooth and spherical surface, with mean particle size around 300 nm. The MMP-1 activity assay and *in vitro* drug release profile of NPs indicated that the bioactivity of the enzyme can be reserved where the encapsulation allows prolonged release of MMP-1 over 60 days. Taken together, we reported here novel PLGA-PCL NPs for sustained release of MMP-1, which may provide an ideal MMP-1 delivery approach for tissue reconstruction therapy.

## Background

Matrix metalloproteinases (MMPs) are a family of enzymes capable of degrading extracellular matrix proteins [1]. This ability is a requirement for cell migration and tissue remodeling, both of which play essential roles in embryonic development, reproduction, tissue remodeling as well as many other physiological and pathological processes. Matrix metalloproteinase-1 (MMP-1), known as interstitial collagenase, plays a pivotal role in degrading collagen and resolving scar to enhance muscle healing [2-4]. Several investigations have shown that direct injection of proMMP-1 into fibrotic skeletal muscle resulted in the reduction of collagen content without adversely affecting the uninjured muscle. More recently, a poly(ethylene glycol) (PEG)-modified form of MMP-1 (PEG-MMP-1) has also been reported, which was prepared by reacting MMP-1 with an amine-reactive PEG to achieve higher stability of enzymes and better efficacy in degrading interfibrillar collagen. However, modification with PEG resulted in the inactivation of MMP-1 towards collagen and did not enhance the stability of the enzyme [5]; therefore, superior formulations are still needed to deliver MMP-1 continuously to maintain the drug concentration and effectiveness for long-term tissue reconstruction therapy.

So far, a number of artificial polymers have been investigated extensively to formulate the biodegradable nano-drug delivery carriers, such as polylactide, poly-L-lactic acid (PLLA), polycaprolactone (PCL) and poly(lactide-co-glycolide) (PLGA) [6,7]. Even though they are biocompatible and biodegradable polymers approved by FDA as safe biomaterials for clinical application, the application of polymer nanoparticles (NPs) is still limited because of polymer crystallization, poor flexible property or low biodegradation rate. For example, because of PLLA's high crystallization and relatively low biodegradation rate, the drug release from the PLLA drug carriers is mainly controlled by drug diffusion, a similar way to the nondegradable drug carriers [8,9]. Recently, poly(lactide-co-glycolide-co-caprolactone) (PLGA-PCL), a novel biodegradable block copolymer, has attracted much attention because it colligates the advantages of PLGA and PCL. Characteristics such as excellent biocompatibility, suitable degradable rate, low glass transition temperature (*T*_g_), good miscibility and great permeability make PLGA-PCL an ideal candidate for sustained drug release delivery systems [10].

In general, the preparation of the NPs is a complicated process where a wide variety of different variables may affect the properties of the final products, including particle size distribution, particle morphology, drug encapsulation efficiency and so on. It is a big challenge to experimentally determine how the properties of the NPs are influenced by potential interactions between preparation factors. In this study, we prepared novel MMP-1-loaded PLGA-PCL NPs using double emulsion and solvent evaporation technique. Rotatable central composite design (RCCD) and response surface methodology (RSM) were used to investigate and optimize the impact of critical factors, namely duration of homogenization, agitation speed and volume ratio of organic solvent phase to external aqueous phase, on the response properties of the yielded NPs, such as mean particle size and entrapment efficiency [11,12]. We first incorporated these multiple factors into mathematical polynomial equation models; second, we solved the equations and analyzed the response surface contour and plots, and lastly, the computational design of the preparation conditions enabled us to achieve the optimized NPs. Additionally, characterization and characteristics of the optimized MMP-1-loaded NPs including particle size distribution, particle morphology, drug encapsulation efficiency, activity assay of encapsulated MMP-1 as well as *in vitro* drug release behavior were carried out. We believe that the optimized novel NPs would be an ideal MMP-1 prolonged release delivery system.

## Methods

### Materials

MMP-1, Tween® 80 (polyoxyethylene sorbitan monooleate), poly(vinyl alcohol) (PVA; MW 14–16 kDa), Pluronic® F127 triblock copolymer and p-aminophenylmercuric (APMA) were purchased from Sigma-Aldrich (Shanghai, China). The thioester substrate Acetyl-Pro-Leu-Gly-[2-mercapto-4-methyl-pentanoyl]-Leu-Gly-OC_2_H_5_ was from Biomol International L.P. (Plymouth Meeting, PA, USA). Poly(L-lactide-co-glycolide-co-caprolactone) (L-lactide/glycolide/caprolactone 70:10:20 molar ratio, inherent viscosity 1.30 dl/g, in chloroform) was obtained from Daigang Biomaterial Co., Ltd. (Jinan, China). MMP-1 enzyme-linked immunosorbent assay (ELISA) kit was from Fengxiang Biotech Co., Ltd. (Shanghai, China). All other reagents were purchased from Sigma-Aldrich.

### Preparation of MMP-1-loaded NPs

PLGA-PCL NPs containing MMP-1 (0.1 μg/mg of NPs) were prepared by double emulsion-solvent evaporation technique based on the method of Liao et al. with modifications [13,14]. Briefly, 20 mg of PLGA-PCL was dissolved in an appropriate amount of dichloromethane and poured into 100 μL of phosphate buffer saline (PBS; pH 7.4) as the inner aqueous phase (W1) containing MMP-1. Then, the inner emulsion (W1/O) was generated by a high-speed IKA Ultra Turrax homogenizer (IKA, Guangzhou, China) operating at 3,000 rpm for 2 min. Next, the inner emulsion was injected into 10 mL of outer aqueous phase (W2), which was composed of aqueous 1.5% PVA (*w*/*v*) and 2% Tween 80. Multiple emulsion (W1/O/W2) was achieved by homogenization with a high-speed homogenizer at selected speed and time following incubation on ice, and then putting on a rota-evaporator under vacuum (500 mHg) for 3 h at room temperature for the complete evaporation of organic solvent, which led the precipitation of copolymer to form solid NPs. Then, the NPs were collected by centrifugation at 10,000 × *g* for 5 min and washed with distilled water three times, followed by freeze-drying using mannitol as cryoprotectant (PLGA-PCL/mannitol 100:30) to get dry powder containing NPs.

### Experimental design

In recent years, RCCD-RSM, a combined analysis technique of mathematics and statistics, has been widely used to evaluate the effects of multiple variables and to optimize complicated chemical and biotechnological processes. RCCD enables several independent variables to be investigated at the same time using a relatively small number of experiments. RSM optimization method can analyze interactions between variables. Therefore, RCCD-RSM can define the interactions between factors, avoid unnecessary experiments and obtain optimized results. Our pilot experiments suggested that the independent variables such as duration time of homogenization, agitation speed of homogenization and volume ratio of organic solvent phase to external aqueous phase were the main factors that affected the particle size distribution and encapsulation efficiency of the NPs. For each independent variable, we took into consideration the feasibility of preparing NPs at extreme values and selected the experimental range and the factor levels based on the results of our pilot experiments, which are listed in Table 1. A total of 20 tests are presented in Table 2. All the formulations in these experiments were prepared in triplicate.

**Table 1:** Independent variables and their corresponding variables of NP preparation for RCCD

**Variables**	**Levels**				
	**−1.682**	**−1**	**0**	**+1**	**+1.682**
*X*_1_ (min)	2	4	7	10	12
*X*_2_ (krpm)	2	2.8	4	5.2	6
*X*_3_	0.1	0.18	0.3	0.42	0.5

*RCCD*, rotatable central composite design; *X*_1_, duration time of homogenization; *X*_2_, agitation speed of homogenization; *X*_3_, volume ratio of organic solvent phase to external aqueous phase.

**Table 2:** RCCD consisting of three experimental factors in coded and actual levels with experimental results

**Formulation**	**Coded independent variables**			**Actual independent variables**			**Dependent variables**	
	***X***_**1**_	***X***_**2**_	***X***_**3**_	***X***_**1**_	***X***_**2**_	***X***_**3**_	**EE (%)**	**Mean size**
1	1	1	1	10	5.2	0.42	36.96	381.2
2	1	1	−1	10	5.2	0.18	28.12	321.1
3	1	−1	1	10	2.8	0.42	42.74	510.2
4	1	−1	−1	10	2.8	0.18	41.09	770.2
5	−1	1	1	4	5.2	0.42	49.08	426.3
6	−1	1	−1	4	5.2	0.18	44.67	371.9
7	−1	−1	1	4	2.8	0.42	61.98	806.7
8	−1	−1	−1	4	2.8	0.18	54.10	1,216.6
9	+1.682	0	0	12	4	0.3	20.14	420.6
10	−1.682	0	0	2	4	0.3	60.50	790.1
11	0	+1.682	0	7	4	0.3	32.38	362.7
12	0	−1.682	0	7	2	0.3	50.14	980.1
13	0	0	+1.682	7	4	0.5	47.22	522.2
14	0	0	−1.682	7	4	0.1	32.19	539.8
15 to 20	0	0	0	7	4	0.3	37.38	471.1

*n =* 3. EE, entrapment efficiency; *RCCD*, rotatable central composite design; *X*_1_, duration time of homogenization; *X*_2_, agitation speed of homogenization; *X*_3_, volume ratio of organic solvent phase to external aqueous phase.

### Characterization of MMP-1-loaded NPs

#### Particle size

The mean diameter of the NPs was measured by dynamic light scattering (DLS) analysis using a NICOMP 380 ZLS zeta potential/particle sizer (PSS Nicomp, Santa Barbara, CA, USA) equipped with a 5-mW helium-neon laser at a wavelength of 633 nm and 90° scattering angle. All samples were diluted with ddH_2_O to produce a suitable scattering intensity before measurement. Each analysis was repeated three times.

#### Surface morphology

The morphological examination of the NPs was performed by scanning electron microscopy (SEM; Carl Zeiss SMT, Oberkochen, Germany) [15]. A drop of the NP suspension was placed on a glass cover slide and dried under vacuum for 12 h. The slides were subsequently mounted onto metal stubs using double-sided adhesive tape and vacuum-coated with a thin layer (100 to 150 Å) of gold. Then, the NPs were examined by SEM at 15 or 20 kV.

#### Encapsulation efficiency

The encapsulation efficiency (E.E.) of the NPs was determined by the cold centrifugation separation of NPs from the aqueous medium containing the non-associated MMP-1, and was calculated as E.E. (%) = [(Total protein amount − Free protein amount)/Total protein amount] × 100%. Briefly, samples were centrifuged (20,000 × *g* at 4 °C for 10 min), and free MMP-1 in the supernatant was determined using the MMP-1 ELISA kit as per manufacturer's protocol: 100 μL of MMP-1 standard solution or MMP-1-containing samples were added into the microtiter plate wells, and then, all unbound sites were blocked to prevent false positive results. Second, 100 μL of biotinylated antibody was added to each well to combine MMP-1; then, 100 μL of prepared streptavidin solution was added. Third, 100 μL of TMB one-step substrate reagent was added to each well to develop color; then, the stop solution was added to change the color from blue to yellow. The intensity of the color in proportion to the amount of MMP-1 is measured at 450 nm. The test procedure was performed for a total of three times for each sample.

### MMP-1 activity assay

To evaluate the influence of the NP preparation procedure on the MMP-1 enzymatic activity, we measured the activity of the encapsulated MMP-1 using Acetyl-Pro-Leu-Gly-[2-mercapto-4-methyl-pentanoyl]-Leu-Gly-OC_2_H_5_ as the thioester substrate. The release of free thiols in the presence of excess 5,50-dithiobis(2-nitrobenzoic acid) (DTNB) was continuously monitored with a Varian Cary 50 ultraviolet spectrophotometer (Varian Inc., Palo Alto Inc., CA, USA) at 412 nm for 30 min at 37 °C. Briefly, 20 mg of dried MMP-1-loaded NPs were first suspended in 1 mL assay buffer (50 mM HEPES, 10 mM calcium chloride, pH 7.5) at 37 °C with a stirring speed of 100 rpm for 3 days. Then, 200 μL of the release medium was withdrawn and centrifuged at 20,000 × *g* for 10 min at 4 °C; the supernatant was analyzed for MMP-1 mass by using a MMP-1 ELISA kit following the manufacturer's protocol. For comparison, non-encapsulated MMP-1 at the same concentration was treated with the same method and used as the 100% bioactivity benchmark. Second, the released MMP-1 in the sample and the non-encapsulated MMP-1 were activated respectively via incubation with APMA (1 mM) for 2 h at 37 °C. The DTNB (1 mM) was then added and pre-incubated at 37 °C for 3 min prior to the addition of thioester substrate (100 μM final concentration). The total reaction volume was 100 μL. One unit of MMP-1 activity was defined as the amount of enzyme required to catalyze substrate hydrolysis at a rate of 1 Abs/min.

### *In vitro* release of MMP-1 from NPs

*In vitro* release profile of MMP-1 from the optimized NPs was performed using dialysis membrane diffusion method [16]. Briefly, 50 mg of dried NPs were re-dispersed in 2 mL PBS (pH 7.4) and placed in a dialysis membrane bag with a cutoff MW of 100,000 Dalton (Spectrum Lab., Rancho Dominguez, CA, USA). The release experiment was initiated by placing the end-sealed dialysis bag into 50 mL PBS buffer (pH 7.4) at 37 °C with a stirring speed of 100 rpm. At chosen time points, 1 mL release medium was withdrawn and replaced with an equal volume of fresh medium. The amount of the released MMP-1 mass was measured by the MMP-1 ELISA kit [17]. Results were expressed as cumulative release of MMP-1 from NPs with three replicates.

### Data analysis

For analyzing the influence of multiple factors, mathematical polynomial equation models of design were established to correlate the independent variables to the dependent variables. The obtained polynomial equation for each response property was analyzed using RSM separately. By solving the regression equation and also by analyzing the response surface contour and surface plots, the optimal levels of the selected independent variables were achieved. Statistical tests were performed with STATISTICA 6.0 software (analysis of variance (ANOVA)) to determine the statistical significance of each model.

## Results and discussion

### Optimization analysis

Preparation of the NPs is a complex procedure as it involves several variables. Even slight changes may have significant impact on the quality of the final products [18,19]. In this study, RCCD and RSM were implemented to optimize the preparation procedure of the NPs, which made it possible to investigate a high number of independent variables at different levels with only a limited number of experiments [20,21]. The chosen independent variables listed in Table 1 from our pilot experiments were taken into account. Table 2 shows the experimental results concerning the two tested variables on mean particle size and encapsulation efficiency. The dependent variables ranged from 321.1 to 1,216.6 nm and from 20.14% to 61.98%, respectively, indicating sensitivity toward the critical factors studied. For analysis of the influence of multiple factors, quadratic equations were formulated to reflect the relationship between the theoretical response and the independent variables. The statistical analysis of the results generated the following mathematical polynomial equations:

(1) $$\begin{array}{l} Y1=4,205.801-211\text{.451 }X_{1}-938.574\;X_{2}-4,210.624\;X_{3}+4.972\;X_{1}{}^{2}+47.589\;X_{2}{}^{2}+1,248.918\;X_{3}{}^{2}\;\\ +22.465\;X_{1}X_{2}+54.028X_{1}\;X_{3}+680.903\;X_{2}X_{3}(R=0\text{.989, }F=23\text{.763, }P=0.001) \end{array}$$

(2) $$\begin{array}{l} Y2=126.860-6.972\;X_{1}-20.600\;X_{2}-63.934\;X_{3}+0.251\;X_{1}{}^{2}+1.802\;X_{2}{}^{2}+141.326\;X_{3}{}^{2}\\ +0.124X_{1}X_{2}-0.625\;X_{1}X_{3}+3.229\;X_{2}X_{3}(R=0.962\text{,}\;F=6.988\text{,}\;P=0.023) \end{array}$$

where *X*_1_*X*_2_ and *X*_3_ represent the duration time of homogenization, agitation speed of homogenization and volume ratio of organic solvent phase to external aqueous phase, and *Y*1 and *Y*2 represent the particle size and the entrapment efficiency. The regression coefficients (*R*) of these equations were found to be 0.989 and 0.962, respectively, indicating the good correlations between independent variables and dependent variables. ANOVA analysis demonstrated that the quadratic equation models are highly statistically significant (*P* < 0.05).

**Figure 1:**
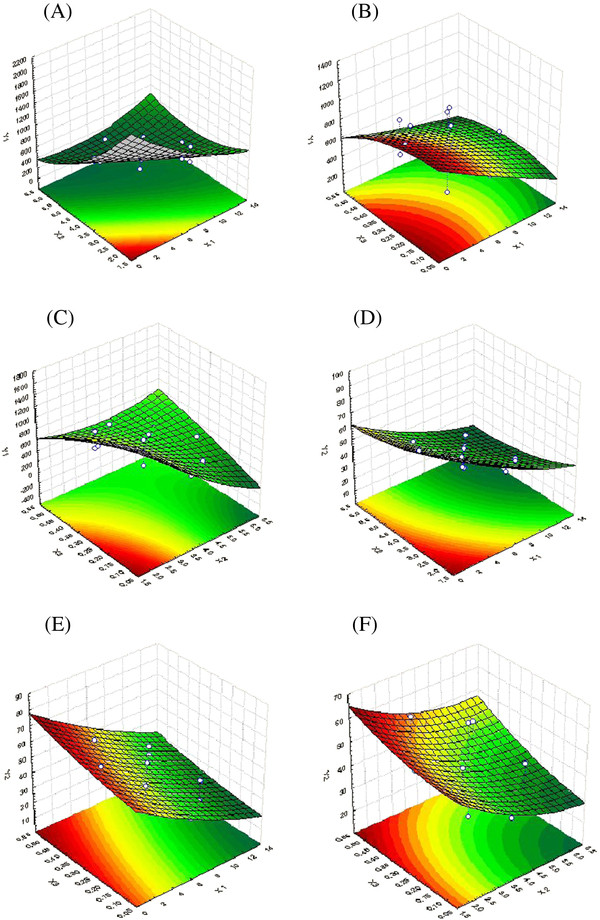
**Response surface plots of mean particle size (*Y*1) and entrapment efficiency (*Y*2) versus three factors. ***X*_1_ = duration of homogenization (min), *X*_2_ = agitation speed (krpm), and *X*_3_ = volume ratio of organic solvent phase to external aqueous phase (*V*/*V*). (**A**, **B**, **C**) The effect of duration of homogenization, agitation speed and volume ratio of organic solvent phase to external aqueous phase on the mean particle size. (**D**, **E**, **F**) The effect of duration of homogenization, agitation speed and volume ratio of organic solvent phase on the (EE)%.

The obtained polynomial equations were further analyzed using RSM, and the three-dimensional surface plots were generated to describe the mathematical models (Figure 1). Figure 1A,B,C displays the effect of duration of homogenization, agitation speed and volume ratio of organic solvent phase to external aqueous phase on the mean particle size. It can be seen from Figure 1A that an increase in the duration and intensity of homogenization within limits can lead to a remarkable decrease in the mean particle size, whereas excessive homogenizations can indeed increase the mean particle size again. The reason may be that excess dispersion can lead to increase in viscosity of the emulsion solution where high viscosity of the emulsion solution slows down the dispersion of organic phase into aqueous phase and leads to the formation of bigger aggregates. Figure 1B shows that increasing the duration of the homogenization and volume ratio of organic solvent phase to external aqueous phase can all produce particles with smaller size. Figure 1C indicates that optimal particle size can be achieved by taking suitable homogenization speed and volume ratio of organic solvent phase to external aqueous phase.

Figure 1D,E,F indicates the effect of duration of homogenization, agitation speed and volume ratio of organic solvent phase to external aqueous phase on entrapment efficiency. Figure 1D shows that an increase in either duration of homogenization or agitation speed all results in proportionate decrease in the entrapment efficiency. Figure 1E,F shows that the increase in volume ratio of organic solvent phase to external aqueous phase can increase the entrapment efficiency, which may be due to the fact that the organic solvent phase acts as a disperse medium. However, when excess organic solvent easily led to phase inversion, we observed that no NPs could be achieved as soon as the amount of organic solvent increased beyond limit in the experiments.

**Figure 2:**
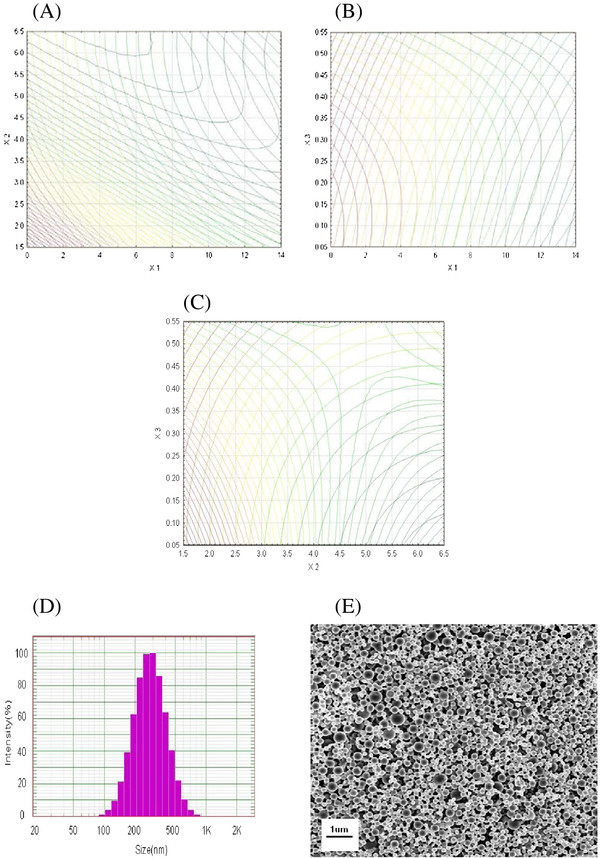
**Optimum zone for NP preparation.** (**A**) Overlapped contour plot for duration of homogenization (min) and agitation speed (krpm); (**B**) overlapped contour plot for duration of homogenization (min) and volume ratio of organic solvent phase to external aqueous phase (*V*/*V*); (**C**) overlapped contour plot for agitation speed (krpm) and volume ratio of organic solvent phase to external aqueous phase (*V*/*V*); (**D**) DLS particle size distributions of MMP-1-loaded NPs (Gaussian distribution); (**E**) scanning electron microscopic images of MMP-1-loaded NPs.

In addition, the optimum zone for NP preparation can be readily found from Figure 2A (overlay of Figure 1A,D), Figure 2B (overlay of Figure 1B,E) and Figure 2C (overlay of Figure 1C,F). The optimized NPs with high entrapment efficiency and small mean diameter were obtained at the duration of homogenization of 7 min, agitation speed of 4.5 krpm and volume ratio of organic solvent phase of 0.4, respectively.

Under the suggested optimal preparation conditions, 309.49 nm of mean particle size and 39.15% of entrapment efficiency were expected to be obtained according to the above equation *Y*1 and *Y*2. The accuracy of the predicted models was further examined by additional independent experiments at the suggested optimal preparation conditions where the yielded NPs had an actual mean particle size and entrapment efficiency of 322.7 ± 18.1 nm and 38.75 ± 4.74% (*n* = 3). The bias values between the actual and predicted values were 4.4% and 1.0%, respectively, which was calculated as (predicted-observed value)/predicted value × 100%. Thus, these demonstrate that by utilizing the mathematical models, optimal NPs can be obtained through a reliable and reasonable manner.

### Nanoparticle size and morphology

The mean diameter and particle size distributions of the optimized NPs were determined by DLS measurement [22]. The optimized NPs had a mean particle size of 322.7 ± 18.1 nm and a polydispersity index below 0.3, indicating a relatively narrow size distribution (Figure 2D).

The SEM image (Figure 2E) shows smooth, homogeneous and spherical-shaped spheres in nano range, and there is no aggregation after lyophillization under the optimal conditions. The NPs have an average particle size of about 300 nm in the range of 100 to 400 nm, which was similar to the data of DLS measurement.

### *In vitro* activity of encapsulated MMP-1

**Figure 3:**
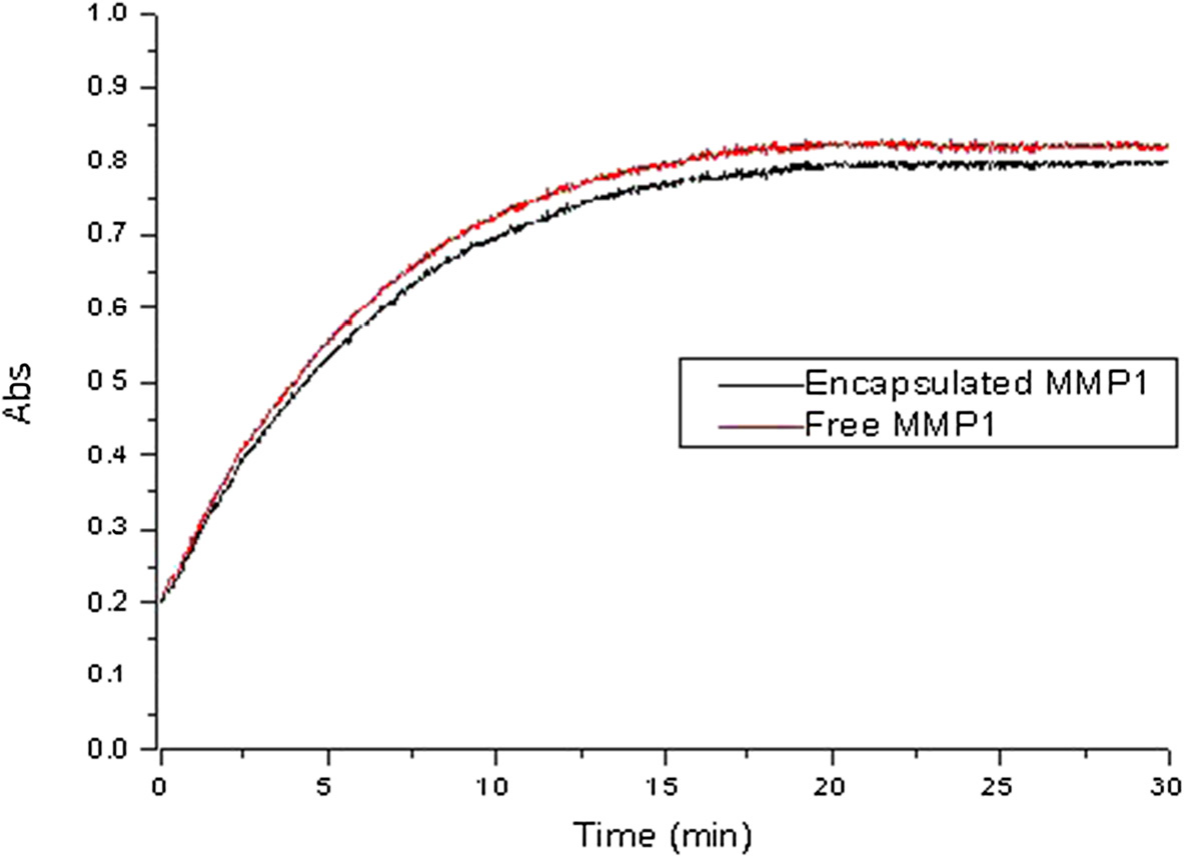
***In vitro *kinetic curve for free and encapsulated MMP-1-catalyzed degradation of thioester peptide substrate.** (*n =* 3).

MMP-1, like all MMPs, is expressed as a zymogen (proMMP-1) and must, therefore, be activated by disrupting the interaction between a cysteine molecule in the enzyme's propeptide domain and a zinc molecule situated in the active site. Latent MMP-1 can be activated *in vitro* by incubation with organomercurial compounds and other chemical agents that modify sulfhydryl groups. *In vivo*, MMP activity is regulated by a group of endogenous proteins, called tissue inhibitor of metalloproteinases, which bind to active and alternative sites of the activated MMP [23]. In our study, the activity of the released MMP-1 in the supernatant and free MMP-1 was measured by monitoring substrate hydrolysis at 412 nm for 30 min at 37 °C. In the initial phase (within the first 3 min), the kinetic curve revealed a linear relationship, whereas the late phase showed a slower and steady reaction progression (Figure 3). We found minimal difference in terms of the enzymatic activity between the encapsulated MMP-1 and free MMP-1 (0.03763 ± 0.0012 vs. 0.03879 ± 0.0008). Thus, our data clearly demonstrated that the preparation procedure had no obvious effect on the MMP-1 enzymatic activity.

### *In vitro* release of optimal NPs

**Figure 4:**
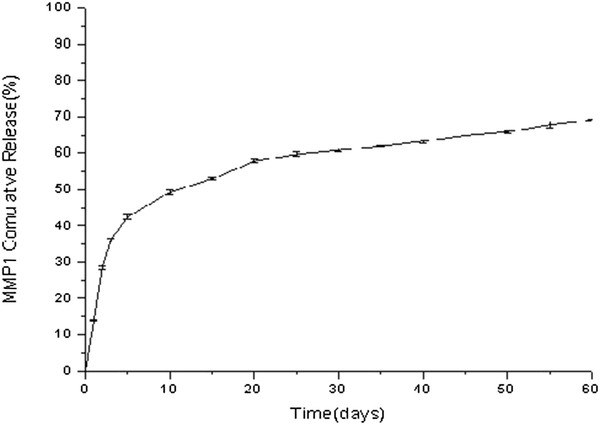
***In vitro *release profile of the optimized MMP-1-loaded NPs.** (*n =* 3).

The NP formulation provided a sustained drug release pattern, which was characterized by a burst release at the initial stage and a sustained release subsequently [24]. Figure 4 shows the cumulative *in vitro * release of MMP-1 from PLGA-PCL NPs where approximately one-third of MMP-1 (36.13 ± 0.39%) was released from the NPs within the first 3 days (burst effect), and the rest (approximately 64%) was continuously but slowly released for the next 60 days. We believed that the initial burst effect was due to the release of the surface-associated protein, whereas the slower phase represented a continuous release of the MMP-1 protein entrapped in the NPs. These results implicate that the optimized NPs can be given as an ideal *in vivo* MMP-1 prolonged release delivery system.

## Conclusions

In the present study, we prepared MMP-1-loaded PLGA-PCL NPs to provide controlled enzyme delivery system. We used RCCD and RSM to investigate the impact of critical factors on the response properties and optimize the conditions to lower the mean size of the particles without jeopardizing its entrapment efficiency. The optimized formulation provided a uniform small particle size with high entrapment efficiencies. Moreover, we characterized the optimized MMP-1-containing PLGA-PCL NPs by DLS and SEM. *In vitro* drug release study showed that the entrapped MMP-1 was released over a period of 60 days in a controlled manner with normal enzymatic activity. Taken together, these NPs are therefore a promising system for prolonged release of enzymes and have great potential application in better control of MMP-1 therapy.

## Competing interests

The authors declare that they have no competing interests.

## Authors’ contributions

PS carried out the overall experiments. HS conceived of the study, participated in its design and revised the manuscript. DC and JQ participated in the device fabrication and gave advice. PS and HS interpreted the results together and drafted the manuscript. MX gave advice on this work. HG conceived of the study and revised the manuscript. All authors read and approved the final version of the manuscript.
